# Motivating Weight Loss Among Black Adults in Relationships: Recommendations for Weight Loss Interventions

**DOI:** 10.1177/10901981221129182

**Published:** 2022-10-15

**Authors:** Candice L. Alick, Carmen Samuel-Hodge, Alice Ammerman, Katrina R. Ellis, Christine Rini, Deborah F. Tate

**Affiliations:** 1 The University of North Carolina at Chapel Hill, Chapel Hill, NC, USA; 2 University of Michigan, Ann Arbor, MI, USA; 3 Northwestern University, Chicago, IL, USA

**Keywords:** weight loss, African American/Black, motivations, triggers, barriers, intervention

## Abstract

**Background:**

Black men and women have the highest rates of obesity in the United States. Behavioral weight loss programs incorporating intimate partners may be effective in combating obesity among this population. Yet, current participation in these programs is low. Identifying motivations and triggers to weight loss may provide insight in designing programs to increase participation.

**Aim:**

To determine triggers and motivations for weight loss among Black men and women in committed relationships to inform development of weight loss interventions.

**Method:**

Twenty semi-structured interviews, based on an integrated theoretical framework of interdependence and communal coping and the social cognitive theory, were conducted among Black heterosexual couples where one or both individuals intentionally lost ≥5% of their body weight in the last 6 months in a metropolitan region in a southern state. Interviews were transcribed and coded. Themes were identified following multi-rater coding and direct and conventional content analysis. Recommendations were developed from emergent themes.

**Results:**

Among individuals with recent weight loss, personal relevancy and awareness (health awareness and self-awareness) were identified as personal triggers for weight loss initiation. Health concerns and appearance were general motivations among the total sample.

**Conclusion:**

Emphasizing the impact of weight on daily functioning and quality of life, and increasing awareness of personal weight status and health consequences, may increase weight loss initiation and participation in weight loss programs among Black men and women in committed relationships. Findings also highlight strategies to improve recruitment and retention and guide intervention development and implementation for this population.

In the United States, nearly 50% of Black adults are affected by obesity (Flegal et al., 2016). Consequentially, Black adults have the highest rates of diagnosed diabetes (Centers for Disease Control and Prevention, 2020), among the highest rates of death caused by heart disease (Van Dyke et al., 2018), and over 50% suffer from high blood pressure (Virani et al., 2020). Behavioral weight loss programs are one of the most effective treatments for obesity and can prevent or delay obesity-related health issues (Smith & Wing, 2000).

In obesity research, the influence of an individual’s environment on weight loss has increased, with focus on the family context (Gorin et al., 2017; Holway et al., 2018; Samuel-Hodge et al., 2010). Family members, specifically cohabitating intimate partners, experience concordance in health statuses and behaviors, leading to intentional and unintentional influences on weight loss (Cobb et al., 2016; Cohen, 2004; Cornelius et al., 2016; Umberson & Karas Montez, 2010). Consequently, in family-based weight loss interventions for adults, enrolled family members are often spouses (Gorin et al., 2017; Kumanyika et al., 2009; McLean et al., 2003; Samuel-Hodge et al., 2017).

This approach could be useful when addressing weight loss among Black adults considering (1) the centrality of familial relationships in Black culture (Kumanyika et al., 2009; Samuel-Hodge et al., 2017), (2) the interrelatedness of health behaviors among family members, and (3) the familial influence on behavior and health outcomes (Holway et al., 2018). Limited data also suggest that aspects of family dynamics and functioning (i.e., cohesion and support) may also be important to weight loss success among Black adults (Kumanyika et al., 2009; Samuel-Hodge et al., 2017). However, representation of Black adults in weight loss interventions is low, Black men considerably less than Black women (Lamson et al., 2022; McLean et al., 2003). The potential benefit of familial inclusion in obesity treatment for Black adults is not possible if Black adults are not present in weight loss interventions (Haughton et al., 2018). Thus, before utilizing these relationship strategies to promote positive health behavior like healthful eating and physical activity, it is important to improve recruitment and enrollment of Black adults, specifically, in committed relationships, into weight loss interventions for familial involvement to be advantageous. Previous studies have found it challenging to recruit Black adults in health-related behavioral research studies (Turner-McGrievy et al., 2021). Improving recruitment messages to increase relevancy of weight loss interventions among Black adults may increase the likelihood of enrollment of this population (Crane et al., 2020).

To address recruitment and enrollment, efforts to identify and describe perceptions of weight and motivations for weight loss among Black men and women in committed relationships are needed. Identifying the relationship between perception of weight and weight loss (Haynes et al., 2018) and triggers for weight loss could inform the design of studies and interventions to increase involvement of Black adult men and women in committed relationships. To our knowledge, no studies have explored perceptions of weight or triggers for weight loss among Black men and women in committed relationships. This study sought to determine the perception of weight and weight management among Black men and women in committed relationships by identifying salient motivators of weight loss from their perspectives and experiences using in-depth interviews. These results informed the content for recruitment messages and development of a spousal support weight loss intervention for Black men in committed relationships (Alick et al., 2018).

## Method

### Participants

A purposive sample of 10 Black heterosexual couples (10 men and 10 women) participated in the study. Participants were adults who self-identified as being in a heterosexual couple in which one or both had intentionally lost 5% to 10% of their body weight in the last 6 months. Couples were eligible to participate if both members: (1) agreed to participate in separate in-depth interviews, (2) self-identified as Black or African American, (3) were 21 years of age or older, and (4) spoke English.

### Design and Approach

This study used in-depth interview methodology to gather detailed perspectives and experiences of weight loss specific to Black men and women in committed relationships. A phenomenological approach with selected techniques from grounded theory allowed the research team to refine interview probes. Invitations (i.e., flyers, letters, and presentations) to participate were distributed online (e.g., Facebook, historically Black fraternities and sororities listservs) and in person (e.g., at fitness centers, shopping centers, churches). Screeners to determine eligibility for participation were completed on the study website. Interviews were conducted on the telephone. After interviews were completed, couples received a $20 incentive. An Institutional Review Board approved study protocols.

We conducted 20 in-depth, semi-structured interviews between June and November 2015. Participants completed a demographic questionnaire and provided informed consent before interviews. One experienced Black investigator (C.A.) conducted all interviews to ensure consistency. Individual phone interviews were conducted without the spouse/partner, typically lasted between 60 and 90 minutes and were audiotaped using digital recorders.

Questions and probes from the interview guide were informed by previous research, extensive literature review, and adapted questionnaires (Lewis et al., 2003, 2006; Rini & Dunkel, 2010) (Table 1). The guide consisted of open-ended questions exploring topics related to personal beliefs about the importance of weight loss or maintaining a healthy weight and directed questions exploring personal experiences with weight loss. Questions reflected constructs of the integrated theoretical framework of interdependence and communal coping (Lewis et al., 2006), and the social cognitive theory (Schunk & Usher, 2012). Digital recording files were transcribed verbatim and uploaded to Dedoose software (SocioCultural Research Consultants, Los Angeles, CA). Transcriptions were read and reviewed at least three times to achieve immersion of data. The lead reviewer and members of the research team created a codebook based on (1) research questions, (2) prior knowledge from weight management research, and (3) memos (C.A., L.A., H.L.). Research team members were either Black or had extensive experience with the target population through research studies. Interviews were the unit of analysis. Two reviewers independently coded two transcripts (L.A., H.L.). Reviewers discussed and reconciled coding, definitions, and decision rules to develop final codebook. The remaining (*n* = 18) transcripts were coded independently (L.A., H.L.). Coding discrepancies were resolved through consensus. The study team met to discuss quote interpretation and group codes into themes (L.H., E.S., J.O., C.E., C.A.). The analytic approach was a combination of directed and conventional content analysis because the purpose of this research was to inform recruitment messages for and design of a weight loss intervention targeting Black heterosexual couples in committed relationships. To increase the trustworthiness and credibility of the study’s interpretations and findings, multiple reviewers (C.A., L.H., J.O., C.E., E.S.) reviewed and interpreted the data to reduce systematic bias (Church et al., 2019). Data saturation was reached after interviewing 20 participants when no new themes emerged.

**Table 1.** Interview Guide Sample Questions.

1. Let’s start the discussion by talking about what makes maintaining a healthy weight/weight loss as a priority or important for you. Probes: • What does healthy mean to you? • Do you think you are healthy? • If not, what are you willing to do to achieve that image of health? • It is important to you because . . . • A healthy weight means . . . • People lose weight because . . . 2. What was the trigger that made you decide to lose weight? Probes: • What does it take to lose weight? Diet? Exercise? • What prevents you from losing weight? • What motivates? • Do people influence your behavior? How? 3. Please tell me about your experience trying to lose weight OR describe your partner’s experience. Probes: • You first tried to lose weight . . . (He/she) • The last time you tried to lose weight . . . (they) • You lost . . . by . . . (he/she) 4. What did your partner do to help you lose weight? OR what did you do to help your partner lose weight? Probes: • If you needed . . . • Together you . . . 5. What sort of things would get you to participate in a weight loss program? Probe • What could be done to recruit you to participate?

## Results

Table 2 presents participant demographics. Participants were on average 40 years of age, weighed 95.9 kg (*SD* = 25.8) and had Class I obesity (body mass index [BMI] of 30 to <35 kg/m^2^, with a mean BMI of 31.6 kg/m^2^ (*SD* = 25.0). Of the participants who reported losing weight in the past 6 months (*n* = 13) (65%), the average weight loss was 7.6 ± 2.8 kg. Three fourths (75%) had at least some college education, and 90% were employed full-time, with 85% reporting an annual household income of $50,000 or greater. On average, couples reported being in a committed relationship (i.e., living with a spouse or partner) for 12.4 years (*SD* = 7.5) and had 2.2 children (*SD* = 2.1).

**Table 2.** Demographic Characteristics of Participants.

Demographics	Total *N* = 20	Men *n* = 10	Women *n* = 10
Age (years), *M* (*SD*)	40.00 (7.62)	41.4 (7.14)	38.50 (8.17)
Weight (kg), *M* (*SD*)	95.89 (25.80)	113.25 (23.51)	78.42 (13.21)
Body mass index (kg/m^2^), *M* (*SD*)	31.59 (25.80)	34.04 (4.47)	29.14 (4.12)
Weight loss in last 6 months (kg), *M* (*SD*)	7.57 (2.77)^a^	8.36 (2.86)^b^	6.65 (2.59)^c^
Education			
Some college (less than 4 years) or associate	5 (25)	3 (30)	2 (20)
College graduate/baccalaureate	5 (25)	4 (40)	1 (10)
Master’s or doctoral	10 (50)	3 (30)	7 (70)
Employment			
Working full-time	18 (90)	10 (100)	8 (80)
Retired, not working	1 (5)	0 (0)	1 (10)
Looking for work	1 (5)	0 (0)	1 (10)
Income			
$50,000 or more, but less than $60,000	3 (15)	2 (20)	1 (10)
$60,000 or more	14 (70)	7 (70)	7 (70)
Prefer not to answer	3 (15)	1 (10)	2 (20)
Tobacco user			
Yes	1 (5)	1 (10)	0 (0)
No	19 (95)	9 (90)	10 (100)
Committed relationship (years), *M* (*SD*)	12.4 (7.51)	11.8 (7.83)	13.0 (7.54)
Children			
Yes	16 (80)	9 (90)	7 (70)
No	4 (20)	1 (10)	3 (30)
No. of children	2.15 (2.06)	2.4 (2.0)	1.9 (2.23)

a *n* = 13. ^b^*n* = 7. ^c^*n* = 6.

Generated codes (Table 3) to address the objective of this study, to identify and describe perception of weight and identify motivation and triggers for weight loss, were grouped conceptually to provide organization of collected data. The following data are presented by concepts relevant to the study objective.

**Table 3.** Summary of Category, Codes, and Concepts From In-Depth Interviews Among Black Men and Women in Committed Relationships About Weight and Weight Loss.

Category	Codes	Concepts
Importance of maintaining a healthy weight or losing weight	Maintaining a healthy weight, Appearance, Doctor Advisement, Specific Medical Reason, Self-Esteem, Age, To Be Healthy, Family, Absence of Physical Health Problems	Optimal daily functioning/life preservation Health risk reduction Self-confidence in physical appearance
General reasons to initiate weight loss	Generalized Reasons, To Be Healthy, Appearance, Age, Hypothetical	One’s physical appearance Attempting to mitigate health conditions Desire to be healthy
Personal trigger for weight loss	Appearance, Trigger for Weight Loss, To Fit Into Clothes, Family, Personal Health Status, Achieving Personal Definition of Health	Not recognizing oneself Recognizing the impact of personal weight on family Having a medical diagnosis/event Not fitting previously worn clothing

### Codes and Concepts: Importance of Maintaining a Healthy Weight or Losing Weight

The main concepts regarding the importance of maintaining a healthy weight or losing weight centered around concern for maintaining optimal daily functioning and life preservation, reducing risk of developing an illness, and having self-confidence in physical appearance (Table 3). These concepts are described below, along with representative quotes.

#### Optimal Daily Functioning/Life Preservation

Participants reported a link between weight and being able to perform daily routines without hindrance. For them, excess weight led to being uncomfortable and prevented or impeded being able to perform simple physical demands of life. Discussions highlighted that having too much extra weight made life more difficult and possibly less enjoyable. One participant reported, “[It] is important that I can maintain my independence and maintain the lifestyle that I like to live.” Another stated,

[A healthy weight allows you to perform] . . . your standard day-to-day activity without struggling. I live on the second floor of my apartment complex, and I don’t want to be out of breath and sweating by the time I get to my door, walking to my car, [or] walking in the mall. I don’t want to be gasping for air.

Participants suggested weight impacts how long one might live and to an extent the level of enjoyment one can experience in life. For example, one participant shared that a healthy weight . . . “[means longer life . . . being happier, being able to chase my granddaughter around, . . . being able to enjoy life. When you’re healthier you can do so much more.”

Participants were familiar with technical weight terminology; however, their perception of healthy weight was subjective. According to one participant,

BMI says [what you are] supposed to be . . . that would be the professional side . . . but a healthy weight . . . is one that I feel comfortable in. . . . I know I’m comfortable [at] 130-135 [lbs]. Anything over, I’m out of breath . . .I can’t fit any clothes.

Similar sentiments from other participants highlighted the difference in what Black men and women in committed relationships believe are expectations from health professionals versus what they themselves value and use as an indicator of healthy weight.

#### Health Risk Reduction

Participants felt weight was also related to health risk. Observing the experiences of others provided evidence of the influence of weight on health status and/or conditions; participants were more aware of the benefit of losing weight. One participant suggested that many health conditions were presenting at earlier ages because of an individual’s weight status,

. . . I see a lot of people my age or younger dying from heart attacks, stress related issues, high cholesterol, [high] blood pressure. . . ; you can attribute it to weight and that made me real conscious about losing weight.

Experiences of family members with weight-related conditions both increased awareness of the benefit of a healthy weight and prompted participants to prioritize maintaining a healthy weight. Several participants desired to prevent diseases associated with weight that were prevalent in their family history. One participant said, “. . . heart disease, being obese, joint issues, I see in my family and it’s because they don’t take care of themselves, they don’t work out, they don’t eat right . . . I’m trying to prevent all of that.”

When you carry extra fat in your midsection . . . it increases [the] likelihood of heart attack and other types of diseases. [I prioritize] . . .keeping a healthy weight to make sure I’m around for my wife and my children . . .

Finally, older participants realized increased difficulty in losing weight with age and its implications for chronic diseases (e.g., diabetes).

#### Self-Confidence in Physical Appearance

In addition to health-related concerns, healthy weight was associated with ideal body appearance. Having a particular physique yielded self-confidence. One participant said,

It has a lot to do with physical appearance and having confidence in how I look and present myself . . . being able to go to the store and find things that fit me well, hav[ing] the ability to shop . . .and not being limited by my size. . . In terms of my own personal and mental health, I feel a lot better when I look better.

For one participant, “being a big guy” was not a problem, but he did not want to be obese. He stated, he preferred to “straddle the line instead of going over [it].” Another participant found satisfaction in not appearing overweight, stating “. . . It’s more not appearing to be overweight . . . like having love handles, having your stomach hanging over your belt.” For Black men and women in committed relationships, the importance of maintaining a healthy weight or losing again was not associated with a technical medical definition of healthy weight, but more acceptance and confidence in appearance: “I will never be able to get to that BMI . . . I just want to get to a healthier goal where if I take off my shirt, I feel comfortable.”

### Codes and Concepts: General Reasons to Initiate Weight Loss

Individuals were asked to provide reasons people in general tend to initiate weight loss. Many of the reasons expressed centered around altering one’s physical appearance or attempting to mitigate health conditions. However, a few expressed the desire to be healthy. According to a participant, “Some people do it because they truly want to be healthy. No other reasons just want to be healthy. Not skinny, not itty bitty skinny but just healthy.”

### Codes and Concepts: Personal Trigger for Weight Loss

For the individuals who lost weight (*n* = 13), they were also asked about the pivotal point that triggered them to initiate weight loss. Participant experiences centered around not recognizing oneself, recognizing the impact of personal weight on family, having a medical diagnosis/event, and not fitting previously worn clothing.

#### Not Recognizing Oneself

Gradual weight gain was unnoticed among Black couples in this study. Much weight loss initiation reported by these Black men and women reporting intentional weight loss within the last 6 months centered around a single moment. Discussions revealed participants were not aware of how their appearance had changed over time and maintained a mental image of themselves from the past. Participants reported not realizing they had gained considerable weight and that a moment of shock triggered weight loss attempts. A participant stated, “‘I was looking through [pictures and thought],’ Who’s that fat man? ‘Oh, that’s me! . . .,’ and that was about it.” Another participant mentioned old photos as a weight loss trigger, “Looking at old pictures versus me now, it’s like, ‘Oh man I want that back.’ The appearance is what made me originally make that change.” Participants were not able to provide information for the reasons physical changes were unnoticed over time.

#### Recognizing the Impact of Personal Weight on Family

Among participants who had lost weight, they indicated family as a trigger for losing weight. “Getting married” and wanting to be as healthy as possible for their partner or being in an existing relationship where their partner inspired them to be better were all mentioned. Being able to be an active and present parent and grandparent were also important. A participant stated,

For my kids . . . [I want] to be a healthy family, where we can play together, . . . where I can go outside and I can play. My dad was always an overweight gentleman . . . He’d only play one game with me and then he would be like “I’m out of breath.” . . . I don’t want that to happen to me and my son. . . . I want to be able to play more than just one game. That’s the main reason.

#### Having a Medical Diagnosis/Event

Medical issues were triggers cited by interviewees who lost weight. Some participants mentioned the following as triggers: experiencing severe cases of high blood pressure, headache, and lupus; being diagnosed with type 2 or gestational diabetes; and having their doctor threatening medication as being necessary for treatment. One woman said, “I had a sister that passed away from Lupus and I have Lupus. What motivates me is to live as long as I can. . .and not . . . die at an early age like she did.” Another said, “I decided that I need to get serious about [my weight] because I had what they call a heart attack last year . . . I went in for a bad cold and they found a small blockage.”

#### Not Fitting Previously Worn Clothing

Several interviewees indicated not being able to fit clothes as a trigger to initiate weight loss. These triggers served as external cues of weight gain and prompted weight loss initiation. One interviewee reported, “When I get where I can’t really fit my clothes, I know it’s time to lose weight. I don’t have to get on [a] scale.” Losing weight was a better alternative to purchasing a new wardrobe.

My clothes stayed the same size, and I didn’t . . . I loved my clothes [but] I’m spending money on babies now, I can’t go buying more clothes. I need to get this weight off.

## Discussion

In comparing perspectives of the importance of a healthy weight, general reasons individuals initiate weight loss, and personal triggers of initiation of weight loss, two major themes emerged: personal relevancy and awareness. These themes suggest that the beliefs and current knowledge of these participants and cues were important motivators in seeking healthy weight status, reflecting the constructs of outcome expectations and reinforcement of the social cognitive theory.

*Personal relevancy* centers around achieving and maintaining physical independence (e.g., from one’s family) and personal satisfaction or being comfortable with oneself. The concepts of optimal daily functioning/life preservation, self-confidence in physical appearance, and recognizing the impact of personal weight on family described previously illustrate the association between weight management and personal relevancy. Individuals acknowledged professional medical standards of weight but were more concerned with comfort and functionality than “numbers.” Participants’ beliefs and current knowledge of the benefits of healthy weight or consequences of unhealthy weight provided motivation for seeking healthy weight status. From this study, weight was relevant because of its impact on personal “day-to-day activity.” General statements of the benefits of weight management were not cited as important factors in initiation of weight loss or maintenance of healthy weight. From these data, only certain knowledge was relevant in initiating behavior change, that is, weight loss.

*Awareness* represents both self-awareness and health awareness. The concepts of not recognizing oneself and not fitting previously worn clothing describe how individual weight loss was triggered by an increase in self-awareness. These events served as cues. Individuals were triggered to lose weight after noticing their physical appearance did not match their current physical perception of themselves or from an objective cue (e.g., needing to purchase new clothing). Weight accumulation over time was unnoticed. The concepts of health risk reduction and having a medical diagnosis/event characterize health awareness. Individuals associated weight with disease prevention and management. The awareness of or knowledge of the link between weight and disease motivated the initiation of weight loss. Family members were cited as being contributors to increased health awareness, whether through direct interaction and conversation or secondhand health experiences. Observing the health experiences of others outside of family also contributed to health awareness. These direct and indirect experiences also served as cues to weight loss initiation. Personal relevancy and awareness have emerged as important concepts to shift the priority of weight among this population.

Similar to our results, health concerns (e.g., medical triggering events) (Borgatti et al., 2021; Holley et al., 2016; Mroz et al., 2018; O’Brien et al., 2007) and alteration of physical appearance (Mroz et al., 2018; O’Brien et al., 2007) have previously been shown as reasons for weight loss initiation in the general population. O’Brien et al. (2007) suggest self-esteem and self-image many influence differences in these primary triggers for weight loss. One study of young adults (ages 18–25) showed that improvement in appearance motivated weight loss more than health concerns (Lanoye et al., 2019). Maintaining or regaining physical functioning, as indicated similarly in our sample, was the most significant trigger for weight loss among individuals with morbid obesity (Md-Yasin et al., 2022). Furthermore, doctor recommendations (Schlicht et al., 2017) and improvements in self-confidence (Holley et al., 2016) have also driven individuals to initiate weight loss.

In the few studies examining motivations for weight loss among Black participants, health concerns also motivated weight loss. One study found Black participants were more likely to engage in weight loss than Whites when prompted by a physician (Boepple et al., 2019). Interestingly, obesity status was not associated with weight lost intention among Black participants when compared with Whites (Assari & Lankarani, 2015). This finding supports our results that Black individuals in committed relationships were not motivated to lose weight to achieve a certain weight or because of their weight classification.

Our findings contribute to the existing weight loss literature by (1) examining motivations of weight loss within the context of a relationship, and (2) identifying initial triggers for weight loss from personal experiences in a population that has not been considered previously. Examining motivations for weight loss within the context of a relationship addresses the interpersonal or social environmental influences on behavior and weight. Weight gain has been associated with entrance into a committed relationship (Kershaw et al., 2014; Tymoszuk et al., 2019). Because weight loss is challenging, and committed relationships are a major influence on weight, such investigations may prove useful in weight loss treatments. Self-relevancy and awareness have not been previously identified as motivations or triggers for weight loss in this population. While weight loss interventions have included increasing knowledge and addressing beliefs about the benefits of weight loss, this study identified explicit knowledge and beliefs and also specific cues to initiate weight loss. Thus, when designing weight loss interventions targeting Black men and women in committed relationships, these findings suggest the following recommendations:

1. Personalizing the impact of weight on daily life—The theme of personal relevancy mainly focused on how weight can impede or enhance how an individual functions each day. Thus, the individualization of weight within the personal context (e.g., playing a game of catch or needing adult children to aid in daily errands) can increase motivation for weight loss. Messaging for recruitment materials should emphasize the association between weight and daily routine, increasing knowledge relevant to this population. Intervention activities may include participants highlighting or discussing how weight changes have impacted physical functioning and day-to-day activities. These instances would also provide opportunities for reflecting on daily life prior to weight loss.
2. Emphasizing the association between weight and health—From this study, health conditions like diabetes and heart attacks were readily associated with weight. However, more proximal health-related issues were just as relevant. For example, the ability to breathe and the reduction of headaches were other relevant health issues. Testimonials on how small changes have led to improvements in health conditions, discontinuing usage of c-pap machines for sleep apnea, or no longer having to take high blood pressure medicine after weight loss increase awareness of the vast benefits of maintaining a healthy weight. Individuals may not fully recognize or are aware of how weight impacts almost every aspect of life. To attract Black men and women in committed relationships to weight loss interventions, programs may need to refine health messages to include nontraditionally publicized associations of health and weight (e.g., erectile dysfunction, breathing, joint pains, sleep, etc.). Providing this new knowledge to individuals unaware of these associations may change an individual’s outcome expectations of healthy weight. Our results suggest an awareness of how weight impacts diabetes, cancer, and hypertension but further suggest that these were not the health conditions that triggered weight loss among this population.
3. Encouraging being present or self-awareness—From these findings, weight accumulation was largely unnoticed among participants. For Black men and women in committed relationships to initiate, achieve, and maintain weight loss, paying attention to weight changes over time is critical. Strategies in interventions must allow participants to consistently be aware of weight changes. To prevent unnoticed weight gain over time, interventions have emphasized weight awareness strategies like daily weighing. Daily weighing is suggested to be important in weight management without the consequence of adverse psychological effects (Wing et al., 2007). Because our results indicate the lack of importance of traditional weight measurements involving numbers, using scales may not be the most effective method in this population. Alternative methods to provide consistent awareness of weight changes, for example, the use of waist beads used in various African cultures among women (Nzoiwu, 2015), should be identified and evaluated to serve as reinforcement for weight management.
4. Tempering usage of medical terms—Our findings suggest that standard medical standards (e.g., BMI) were not relevant. Messaging and intervention components should utilize language or terms that resonate with Black men and women. For example, promoting individual weight ranges to enhance quality of life compared with classifying individuals as overweight or obese based on BMI may prove more effective.

While providing invaluable qualitative information, the study had several limitations, including a non-random sample of couples. Couples self-identified as heterosexual, were highly educated, and were recruited from a southeastern metropolitan area. These findings may not hold among couples who are not heterosexual, who are less educated, and who reside in non-metropolitan areas. Second, this sample may not be representative of those who were unsuccessful in losing weight or who may be uncomfortable discussing their weight loss. Finally, these findings may not be representatives of individuals not in a committed relationship. We acknowledge the limitations of this study; however, these findings nevertheless inform culturally relevant intervention design for weight loss interventions.

Strengths of this study included recruiting a sample of both men and women who had successfully lost weight, and, to our knowledge, providing the first qualitative investigation exploring the importance of weight and weight loss from both the male and female perspective and experience as part of a committed couple. Also, this analysis focused exclusively on Black couples; weight-related health disparities in this population support the need for increased efforts to identify mechanisms influencing their weight loss. Finally, our sample consists of participants from a range of ages, years in a committed relationship, and body weight. The similarities reported in experiences and perspectives permit confidence in developing messages to recruit and design interventions and in generating information (e.g., content for group activities, and messages and recommendations to support partners) to enhance couples focused weight loss interventions for Black men and women in committed relationships.

## Conclusion

Findings from this study highlight motivators for weight loss initiation among Black men and women in committed relationships. Both men and women who lost weight reported that their intention was to observe improvements in their well-being, rather than losing a specific amount of weight. The emergent themes of “personal relevancy” and “awareness” provide important information to improve strategies to engage Black heterosexual couples in weight loss. The results from our analysis have informed the design and implementation of a spousal support weight loss program for Black men (Alick et al., 2018). The reasons and triggers Black men and women reported regarding weight loss were used as messages throughout recruitment strategies, program materials, and dialogue. These findings contribute to the existing literature by addressing culturally relevant motivations for weight loss from the perspectives of successful weight loss initiators and losers. These findings further suggest the importance of knowledge, beliefs, and cues critical in initiating weight loss. Our results will improve approaches to address weight management and ultimately impact weight-related health illnesses.
